# Supplementary material for: A role for Tau protein in maintaining ribosomal DNA stability and cytidine deaminase-deficient cell survival
Source: Nat Commun. 2017 Sep 25;8:693. doi: 10.1038/s41467-017-00633-1 (PMC5612969; doi:10.1038/s41467-017-00633-1)
Supplement: Supplementary file 1 — Supplementary Information [file 41467_2017_633_MOESM1_ESM.pdf]

Description of Supplementary Files

File Name: Supplementary Information
Description: Supplementary Figures and Supplementary Tables

File Name: Supplementary Data 1
Description: List of 959 candidates meeting the synthetic lethal screening criteria of 90% increase in death rates in BS-Ctrl(BLM) cells.

File Name: Supplementary Data 2
Description: List of genes differentially expressed between BS-Ctrl(BLM) and BS-BLM cells. Genes upregulated in BS-Ctrl(BLM) cells are shown in red, and those downregulated in these cells are shown in green. $|\text{fold-change}|>1.5$ and $P < 0.05$.

File Name: Supplementary Data 3
Description: List of patients ID along with the expression datasets for CDA and MAPT transcripts in METABRIC breast cancer dataset.

File Name: Supplementary Data 4
Description: List of patients ID along with the expression datasets for CDA and MAPT transcripts in kidney renal papillary cell carcinoma dataset.

File Name: Supplementary Data 5
Description: List of patients ID along with the expression datasets for CDA and MAPT transcripts in kidney renal clear cell carcinoma dataset.

File Name: Supplementary Data 6
Description: List of patients ID along with the expression datasets for CDA and MAPT transcripts in prostate adenocarcinoma dataset.

File Name: Supplementary Data 7
Description: List of patients ID along with the expression datasets for CDA and MAPT transcripts in pheochromocytoma and paraganglioma dataset.

File Name: Peer Review File

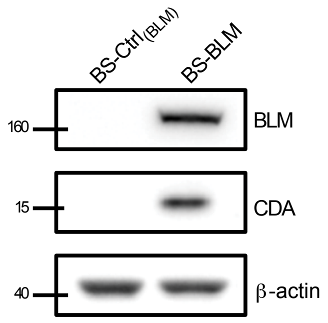

Supplementary Figure 1 | Genome-wide shRNA screen in BS cells. BLM and CDA protein levels determined by western blotting in BS-Ctrl$_{(BLM)}$ and BS-BLM cells. β-actin was used as a loading control.

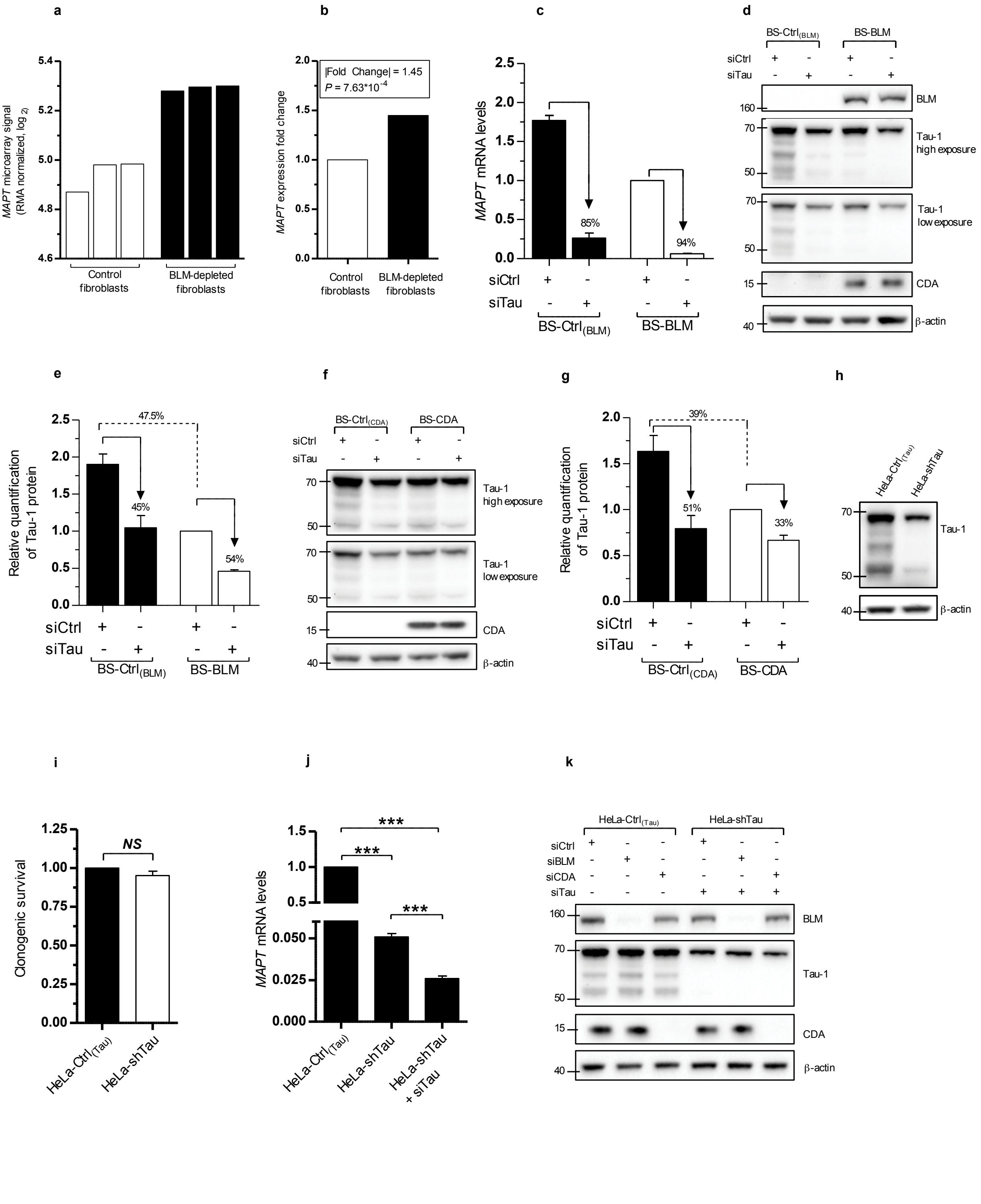

Supplementary Figure 2 | Tau silencing reduces the survival of CDA-deficient cells. (a) *MAPT* mRNA levels in control and BLM-depleted fibroblasts, determined from the microarray expression data available under accession no. GSE54502. The signal was $\log_2$-transformed and normalized with the Robust Multi-array Average (RMA) method. **(b)** Fold-change of *MAPT* expression in BLM-depleted fibroblasts relative to normal fibroblasts. **(c-g)**

Cells were first transfected with either non-targeting or Tau siRNA. Twenty-four hours later, cells were again transfected with the indicated siRNAs. Two days after the second round of transfection, the cells were harvested and *MAPT* mRNA levels were determined by qPCR **(c)**, or BLM, Tau and CDA protein levels were determined by western blotting with BLM, Tau-1 and CDA antibodies, respectively **(d, f)**. **(e, g)** Tau protein quantification. The results are normalized against those for BS-BLM **(e)**, or BS-CDA **(g)**, which were set to 1. **(h)** Tau protein levels determined by western blotting in HeLa-$Ctrl_{(Tau)}$ and HeLa-shTau cells using Tau-1 antibody. **(i)** Clonogenic survival assay. HeLa-$Ctrl_{(Tau)}$ and HeLa-shTau cells were plated in triplicate at three dilutions (200, 400 and 800) in 12-well plates. Seven days later, colonies were fixed and stained with crystal violet. The results were normalized against those for the control cell line, set to 1. **(j)** *MAPT* mRNA levels determined by qPCR in HeLa-$Ctrl_{(Tau),}$ HeLa-shTau and HeLa-shTau + siTau cells. **(k)** HeLa-$Ctrl_{(Tau)}$ and HeLa-shTau cells were first transfected with the indicated siRNAs. Twenty-four hours later, cells were again transfected with the same siRNAs. Two days after the second round of transfection, cells were harvested and BLM, Tau-1 and CDA protein levels were determined by western blotting. For qPCR, mean values for 3 independent experiments are expressed as a fold-change. *B2M*, *β-actin* and *TBP* were used as reference genes for qPCR normalization. Error bars represent the ± SEM of at least three independent experiments. The significance of differences was assessed in two-tailed paired Student's *t*-tests. For western blots, β-actin was used as a loading control. *** $P<0.0005$, *NS*, not significant.

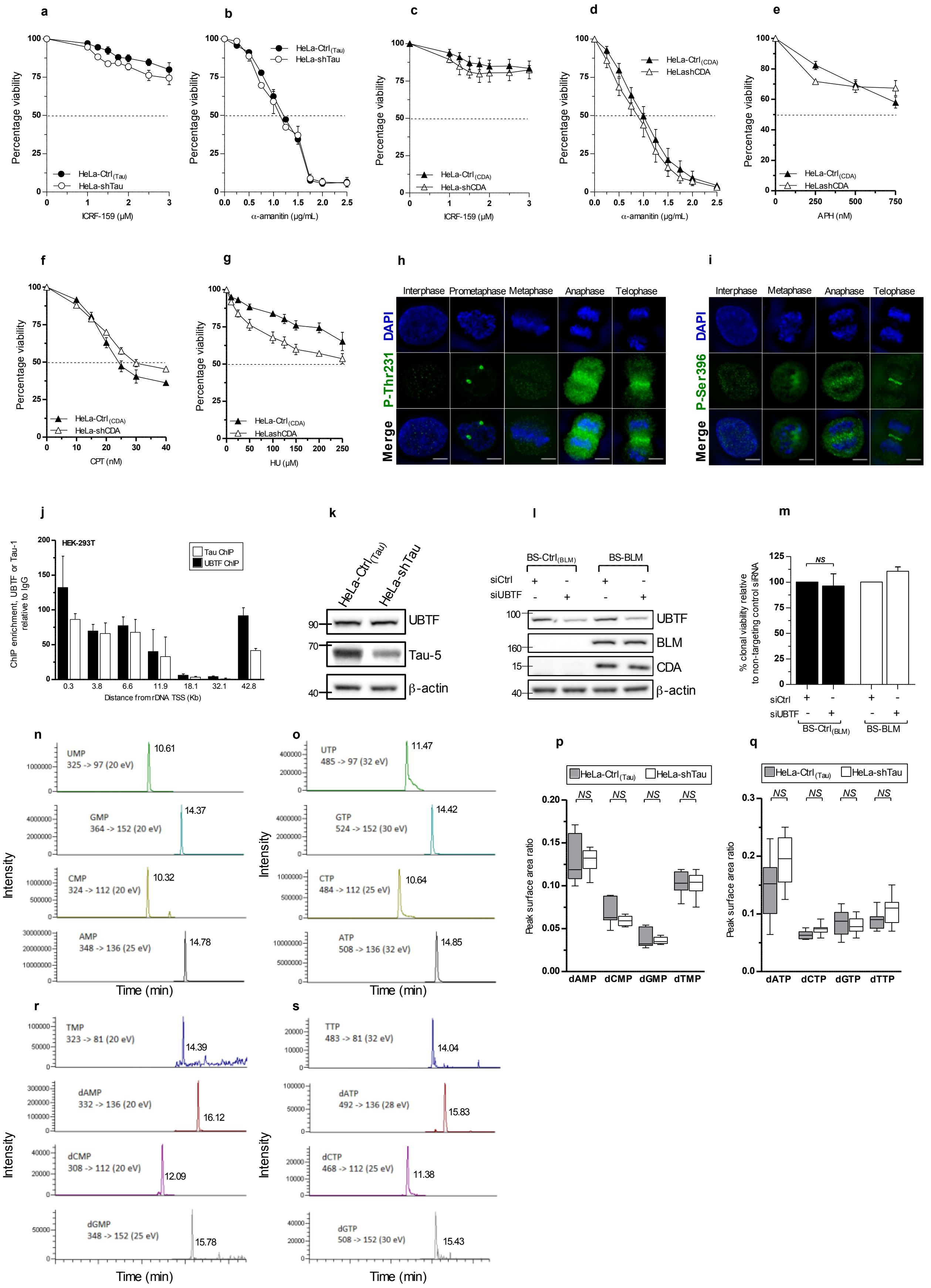
a
Percentage viability
HeLa-Ctrl(Tau)
HeLa-shTau
ICRF-159 (μM)
b
HeLa-Ctrl(Tau)
HeLa-shTau
α-amanitin (μg/mL)
c
HeLa-Ctrl(CDA)
HeLa-shCDA
ICRF-159 (μM)
d
HeLa-Ctrl(CDA)
HeLashCDA
α-amanitin (μg/mL)
e
HeLa-Ctrl(CDA)
HeLashCDA
APH (nM)
f
HeLa-Ctrl(CDA)
HeLa-shCDA
CPT (nM)
g
HeLa-Ctrl(CDA)
HeLashCDA
HU (μM)
h
Interphase
Prometaphase
Metaphase
Anaphase
Telophase
DAPI
P-Thr231
Merge
i
Interphase
Metaphase
Anaphase
Telophase
DAPI
P-Ser396
Merge
j
HEK-293T
Tau ChIP
UBTF ChIP
ChIP enrichment, UBTF or Tau-1 relative to IgG
Distance from rDNA TSS (Kb)
k
HeLa-Ctrl(Tau)
HeLa-shTau
UBTF
Tau-5
β-actin
l
BS-Ctrl(BLM)
BS-BLM
siCtrl
siUBTF
UBTF
BLM
CDA
β-actin
m
NS
% clonal viability relative to non-targeting control siRNA
siCtrl
siUBTF
BS-Ctrl(BLM)
BS-BLM
n
UMP 325 -> 97 (20 eV)
GMP 364 -> 152 (20 eV)
CMP 324 -> 112 (20 eV)
AMP 348 -> 136 (25 eV)
Intensity
Time (min)
o
UTP 485 -> 97 (32 eV)
GTP 524 -> 152 (30 eV)
CTP 484 -> 112 (25 eV)
ATP 508 -> 136 (32 eV)
Intensity
Time (min)
p
HeLa-Ctrl(Tau)
HeLa-shTau
NS
Peak surface area ratio
dAMP
dCMP
dGMP
dTMP
q
HeLa-Ctrl(Tau)
HeLa-shTau
NS
Peak surface area ratio
dATP
dCTP
dGTP
dTTP
r
TMP 323 -> 81 (20 eV)
dAMP 332 -> 136 (20 eV)
dCMP 308 -> 112 (20 eV)
dGMP 348 -> 152 (25 eV)
Intensity
Time (min)
s
TTP 483 -> 81 (32 eV)
dATP 492 -> 136 (28 eV)
dCTP 468 -> 112 (25 eV)
dGTP 508 -> 152 (30 eV)
Intensity
Time (min)

Supplementary Figure 3 | Tau interacts with transcriptionally active rDNA clusters and regulates rDNA transcription. **(a-g)** Survival assays. HeLa-Ctrl$_{(Tau)}$ and HeLa-shTau cells were exposed, for 72 h, to ICRF-159 **(a)** and α-amanitin **(b)**. HeLa-Ctrl$_{(CDA)}$ and HeLa-shCDA cells were exposed, for 72 h, to ICRF-159 **(c)**, α-amanitin **(d)**, aphidicolin **(e)**, camptothecin **(f)** and hydroxyurea **(g)**. Each data point is the mean of at least three independent experiments performed in triplicate. Error bars represent ± SEM. **(h-i)** Immunofluorescence microscopy showing the phospho-dependent Tau antibodies Thr231 (green) **(h)** or Ser396 (green) **(i)** labeling in BS cells. DNA was visualized by DAPI staining (blue). Merged images are shown in the bottom panel. Scale bar: 5 µm. **(j)** Chromatin immunoprecipitations with Tau-1, UBTF and IgG antibodies in HEK-293T cells. DNA was quantified by qPCR with specific primer sets. Data are normalized against non-specific genomic DNA and relative to IgG. Data are the means from three independent ChIP experiments. Error bars represent ± SD. **(k)** UBTF and Tau-5 protein levels determined by western blotting in HeLa-Ctrl$_{(Tau)}$ and HeLa-shTau cells. **(l,m)** Cells were transfected with either non-targeting or UBTF siRNA. Two days after the transfection, the cells were harvested for western blotting **(l)**, or were plated, in a serial dilution series, in 12-well plates. Ten to twelve days later, colonies were fixed and stained with crystal violet. Non-targeting siRNA was used as a control, with values set to 1 **(m)**. **(n-s)** Nucleotide pools were measured in HeLa-Ctrl$_{(Tau)}$ and HeLa-shTau cells. **(n,o,r,s)** Representative ion chromatograms from cell extract. Multiple reaction monitoring (MRM) transitions and collision energy are mentioned for each nucleotide. **(p,q)** Values of peak surface area ratios between the endogenous nucleotide and its internal standard are shown. The data are the means from nine independent measurements corresponding to three independent experiments performed in triplicate. The significance of differences was assessed in Mann-Whitney tests. *NS*, not significant.

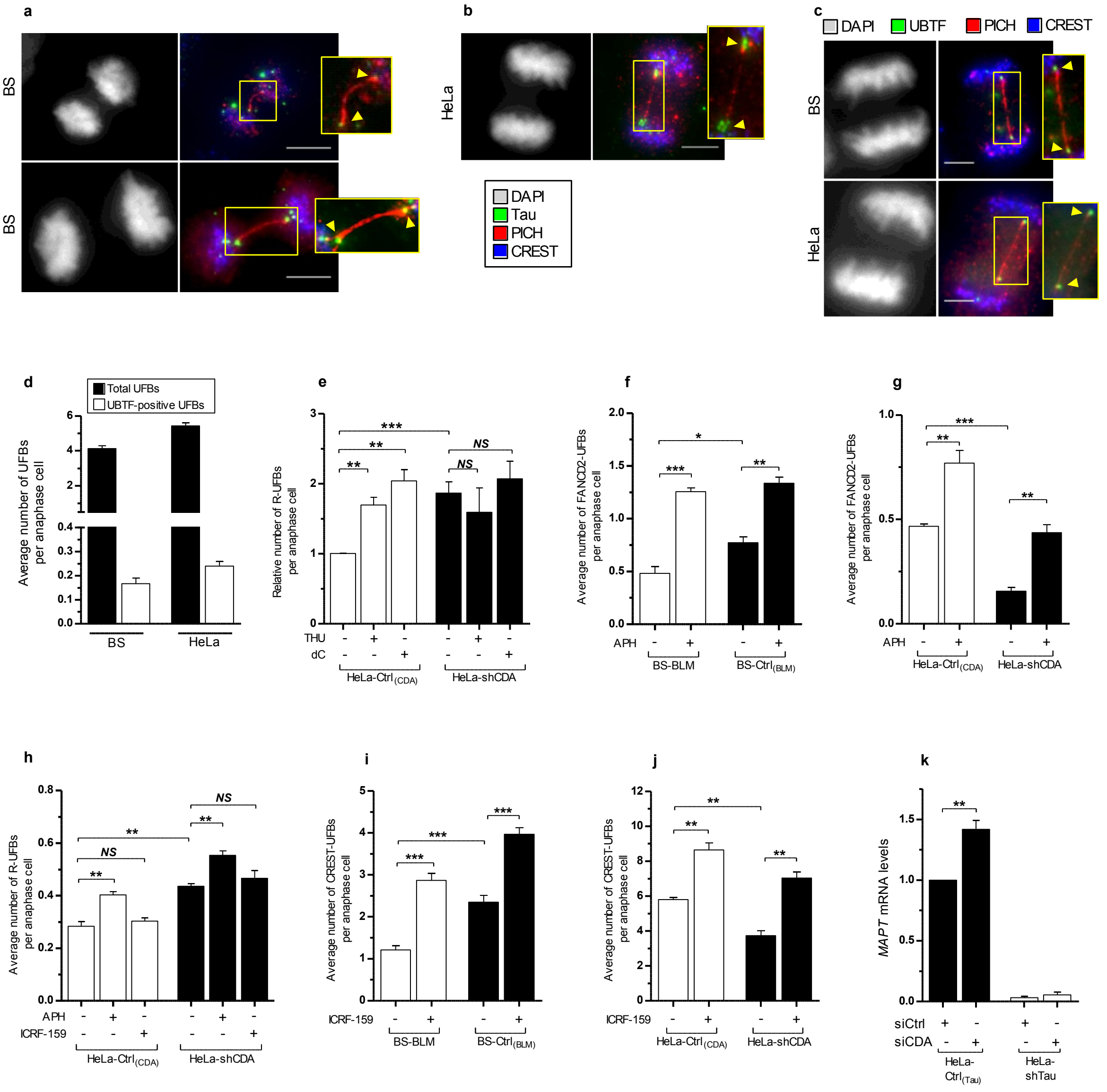

Supplementary Figure 4 | Tau loss alters the genetic integrity of rDNA and CDA-deficient cells. (a-c) Representative immunofluorescence *z*-projection images showing paired Tau foci (green) linked by PICH-positive UFBs in BS **(a)** and HeLa **(b)** anaphase cells, or paired UBTF foci (green) linked by PICH-positive UFBs in BS (top) and HeLa (bottom) anaphase cells. DNA was visualized by DAPI staining (white). Centromeres were stained with CREST serum (blue) and UFBs were stained with PICH antibody (red). In the enlarged images, Tau foci at the extremities of UFBs are indicated by yellow arrows. Scale bar: 5 µm**.** **(d)** Mean number of total and UBTF-positive UFBs per anaphase in BS and HeLa cells. **(e)**

Relative number of R-UFBs per anaphase cell in HeLa-$Ctrl_{(CDA)}$ and HeLa-shCDA cells left untreated or treated with 100 µM tetrahydrouridine (THU) for 2x48 h or 1 mM of deoxycytidine (dC) for 10 h. **(f,g)** Mean number of FANCD2-associated UFBs per anaphase cell in BS-$Ctrl_{(BLM)}$ and BS-BLM cells **(f)**, and in HeLa-$Ctrl_{(CDA)}$ and HeLa-shCDA cells **(g)** left untreated or treated with 0.4 µM APH. **(h)** Mean number of R-UFBs per anaphase cell in HeLa-$Ctrl_{(CDA)}$ and HeLa-shCDA cells left untreated or treated with 0.4 µM APH or 1 µM ICRF-159 for 24 h. **(i,j)** Mean number of CREST-associated UFBs per anaphase cell in BS-$Ctrl_{(BLM)}$ and BS-BLM cells **(i)**, and in HeLa-$Ctrl_{(CDA)}$ and HeLa-shCDA cells **(j)** left untreated or treated with 1 µM ICRF-159 for 24 h. For UFB experiments, at least 200 anaphase cells per condition are counted. The significance of differences was assessed in two-tailed unpaired Student's *t*-tests. **(k)** HeLa-$Ctrl_{(Tau)}$ and HeLa-shTau cells were first transfected with either non-targeting or CDA siRNA. Forty-eight hours later, cells were again transfected with the indicated siRNAs. Three days after the second round of transfection, cells were harvested and *MAPT* mRNA levels were quantified by qPCR. The mean value of 3 independent experiments is represented as a fold-change. *B2M*, *β-actin* and *TBP* were used as reference genes for qPCR normalization. Error bars represent the ± SD of at least three independent experiments. The significance of differences was assessed in two-tailed paired Student's *t*-tests. ** $P<0.005$, * $P<0.05$, *NS*, not significant.

Figure 2b

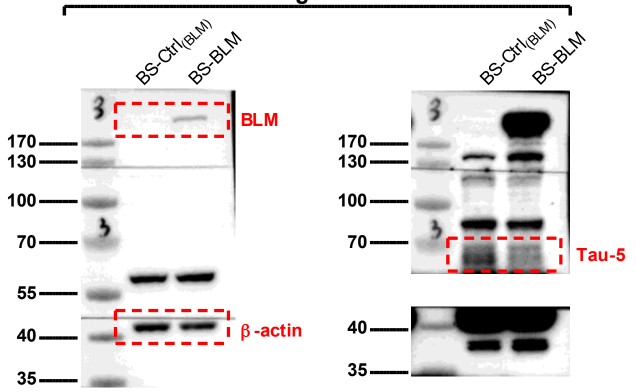

10 sec exposure 600 sec exposure

Figure 2c

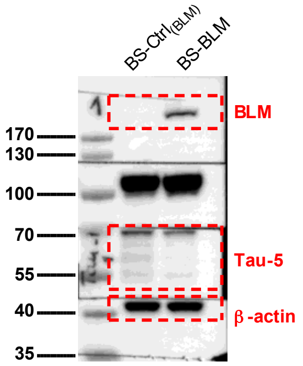

60 sec exposure

Figure 2f

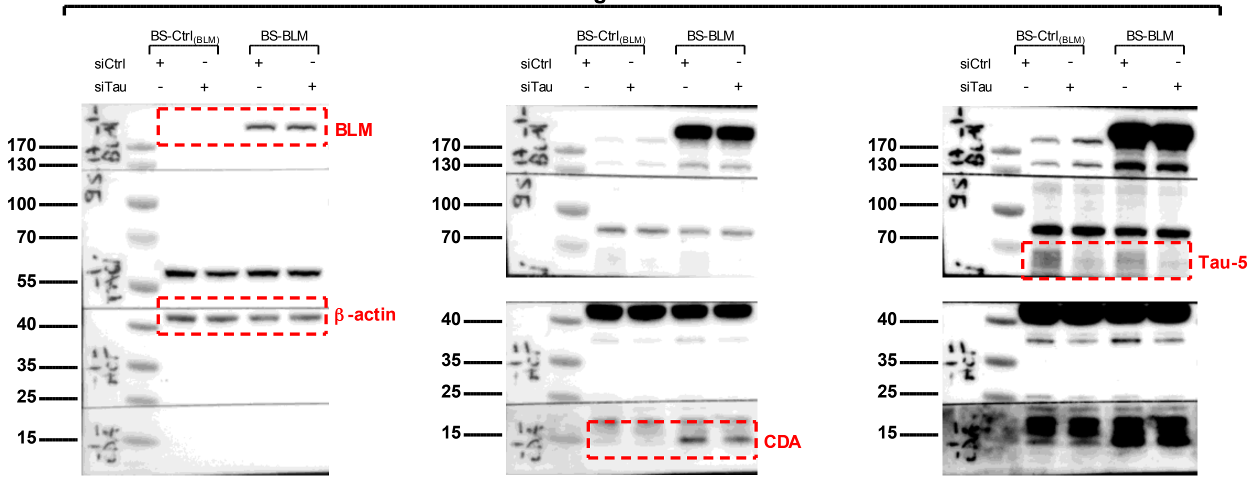

35 sec exposure 300 sec exposure 900 sec exposure

Figure 2i

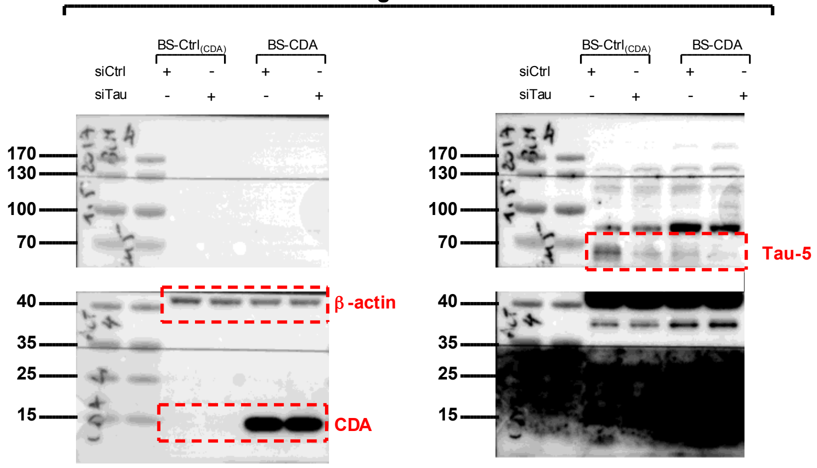

25 sec exposure 600 sec exposure

Figure 2m + Supplementray Figure 3k

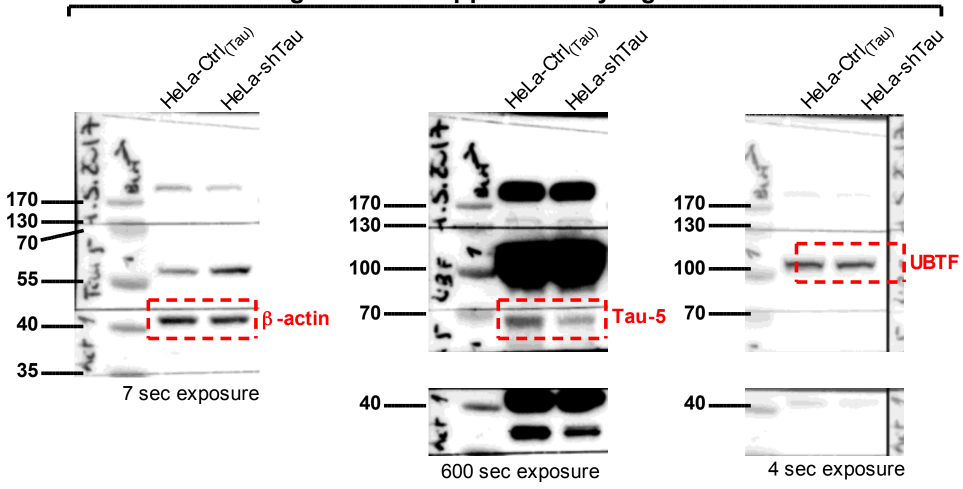

Figure 2n

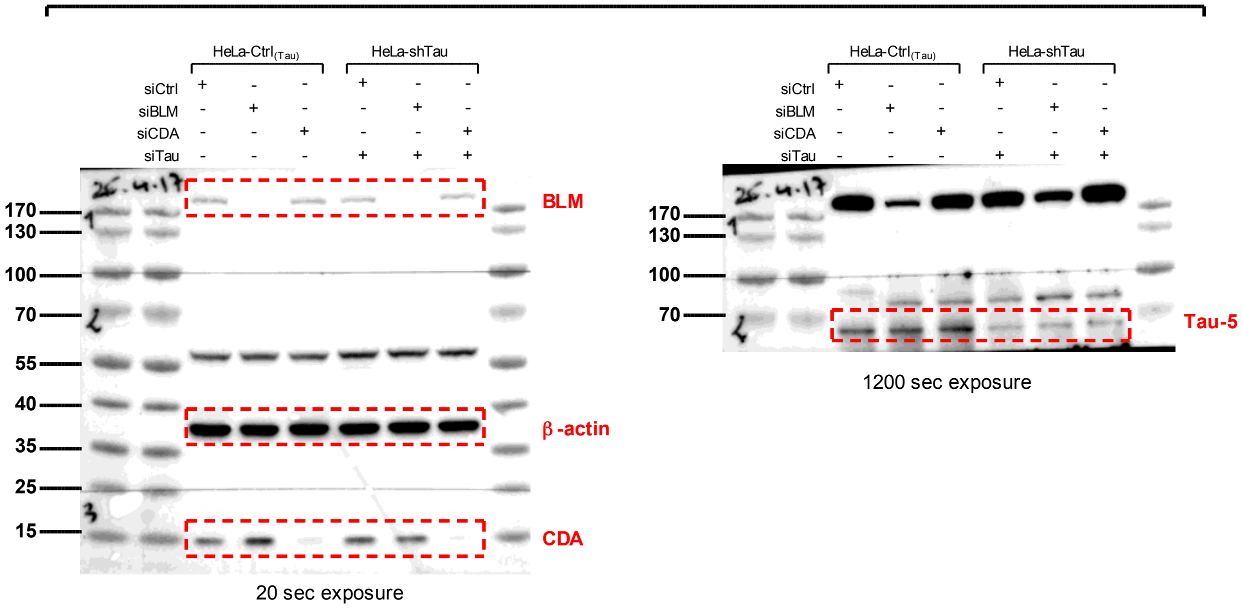

Figure 3b

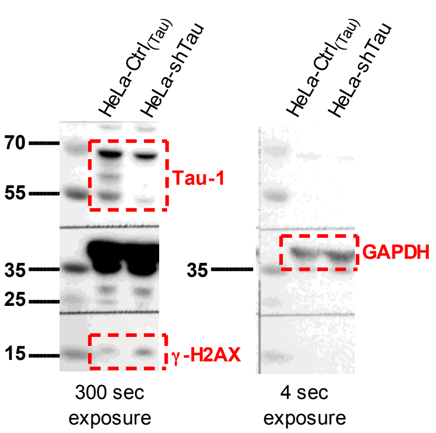

Figure 3j

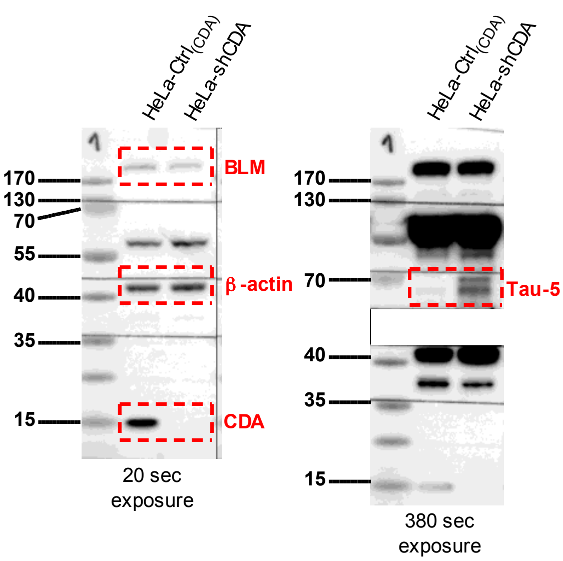

Supplementary Figure 3l

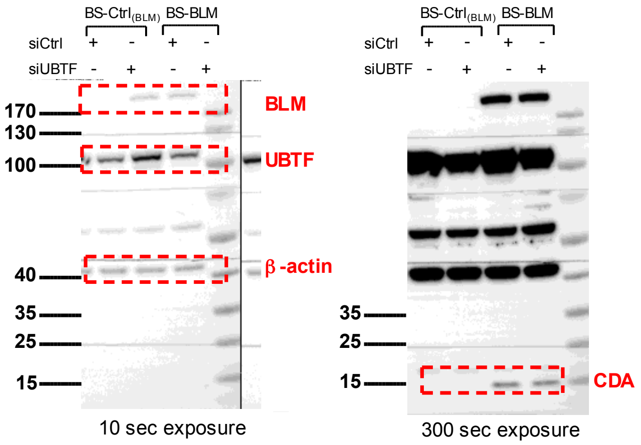

Supplementary Figure 6a

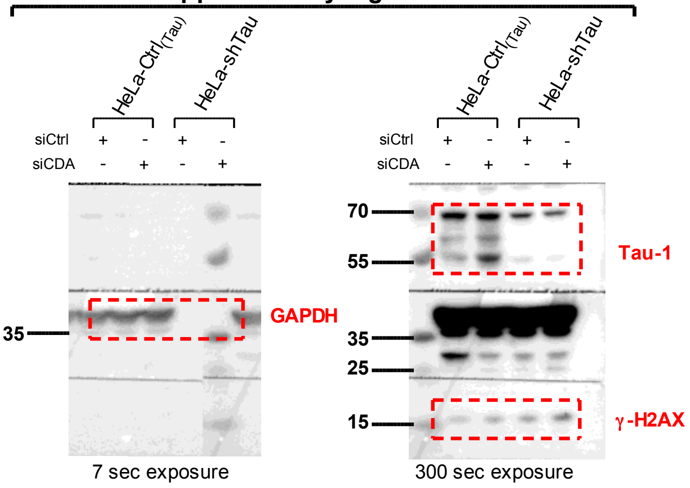

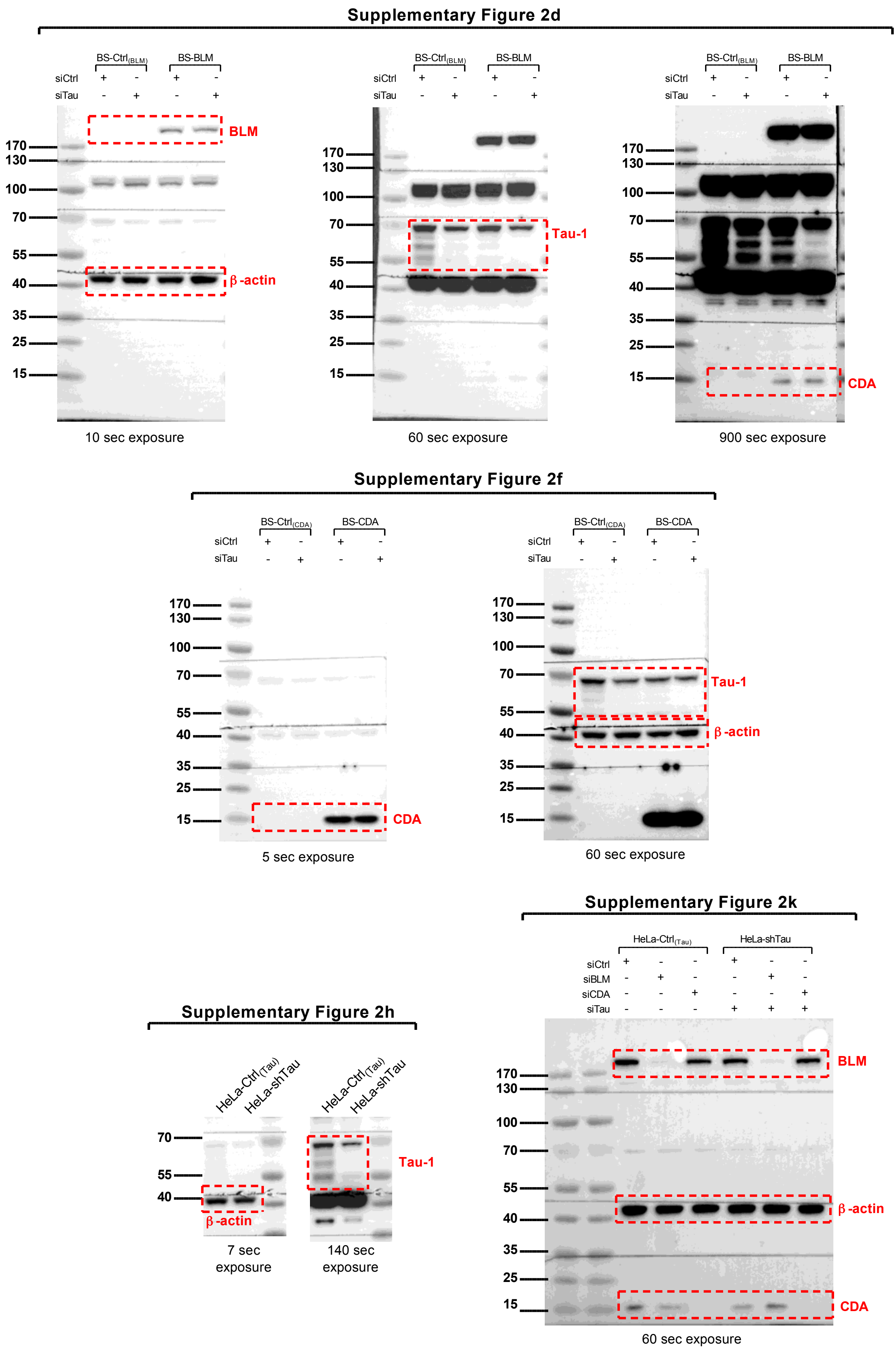

Supplementary Figure 5 | Pictures of full uncropped immunoblots.

Annotation Cluster 1	Enrichment Score: 2,86				
**Category**	**Term**	**Count**	**%**	***p* value**	**Fold Enrichment**
SP_PIR_KEYWORDS	atp-binding	92	10,42	4,96E-5	1,52
SP_PIR_KEYWORDS	nucleotide-binding	108	12,23	2,47E-4	1,41
UP_SEQ_FEATURE	nucleotide phosphate-binding region:ATP	66	7,47	1,04E-3	1,50
GOTERM_MF_FAT	GO:0005524~ATP binding	103	11,66	1,13E-3	1,35
GOTERM_MF_FAT	GO:0032559~adenyl ribonucleotide binding	104	11,78	1,18E-3	1,35
GOTERM_MF_FAT	GO:0032553~ribonucleotide binding	123	13,93	1,49E-3	1,30
GOTERM_MF_FAT	GO:0032555~purine ribonucleotide binding	123	13,93	1,49E-3	1,30
GOTERM_MF_FAT	GO:0030554~adenyl nucleotide binding	107	12,18	2,16E-3	1,31
GOTERM_MF_FAT	GO:0000166~nucleotide binding	145	16,42	2,20E-3	1,25
GOTERM_MF_FAT	GO:0017076~purine nucleotide binding	126	14,27	2,58E-3	1,27
GOTERM_MF_FAT	GO:0001882~nucleoside binding	108	12,23	3,15E-3	1,30
GOTERM_MF_FAT	GO:0001883~purine nucleoside binding	107	12,12	3,57E-3	1,29

Supplementary Table 1 | DAVID gene ontology analysis for the 959 synthetic lethal candidates.

Biological terms in the leading enriched functional clusters. $P < 0.05$.

Category	Term	Count	%	PValue	Fold Enrichment
GOTERM_BP_FAT	GO:0009152~purine ribonucleotide biosynthetic process	16	1,79	8,79E-04	2,68
GOTERM_BP_FAT	GO:0009260~ribonucleotide biosynthetic process	16	1,79	1,60E-03	2,53
GOTERM_BP_FAT	GO:0009150~purine ribonucleotide metabolic process	17	1,90	1,78E-03	2,41
GOTERM_BP_FAT	GO:0009165~nucleotide biosynthetic process	20	2,23	3,07E-03	2,11
GOTERM_BP_FAT	GO:0031401~positive regulation of protein modification process	20	2,23	3,26E-03	2,09
GOTERM_BP_FAT	GO:0009259~ribonucleotide metabolic process	17	1,90	3,39E-03	2,26
GOTERM_BP_FAT	GO:0006164~purine nucleotide biosynthetic process	17	1,90	3,62E-03	2,25
GOTERM_BP_FAT	GO:0034654~nucleobase, nucleoside, nucleotide and nucleic acid biosynthetic process	20	2,23	4,60E-03	2,03
GOTERM_BP_FAT	GO:0034404~nucleobase, nucleoside and nucleotide biosynthetic process	20	2,23	4,60E-03	2,03
GOTERM_BP_FAT	GO:0006396~RNA processing	43	4,80	5,30E-03	1,54
GOTERM_BP_FAT	GO:0007010~cytoskeleton organization	36	4,02	5,31E-03	1,62
GOTERM_BP_FAT	GO:0051247~positive regulation of protein metabolic process	23	2,57	6,55E-03	1,85
GOTERM_BP_FAT	GO:0006163~purine nucleotide metabolic process	19	2,12	6,82E-03	2,00
GOTERM_BP_FAT	GO:0051640~organelle localization	12	1,34	7,01E-03	2,55
GOTERM_BP_FAT	GO:0032270~positive regulation of cellular protein metabolic process	22	2,46	8,18E-03	1,85
GOTERM_BP_FAT	GO:0016071~mRNA metabolic process	31	3,46	8,23E-03	1,64
GOTERM_BP_FAT	GO:0051240~positive regulation of multicellular organismal process	22	2,46	1,34E-02	1,77
GOTERM_BP_FAT	GO:0044271~nitrogen compound biosynthetic process	27	3,02	1,55E-02	1,63
GOTERM_BP_FAT	GO:0009205~purine ribonucleoside triphosphate metabolic process	13	1,45	1,62E-02	2,18
GOTERM_BP_FAT	GO:0031399~regulation of protein modification process	25	2,79	1,63E-02	1,66
GOTERM_BP_FAT	GO:0009199~ribonucleoside triphosphate metabolic process	13	1,45	1,72E-02	2,16
GOTERM_BP_FAT	GO:0007017~microtubule-based process	22	2,46	1,93E-02	1,70

Supplementary Table 2 | DAVID gene ontology analysis for the 959 synthetic lethal candidates.

Enriched significant biological process terms obtained. $P < 0.05$.

Category	Term	Count	%	PValue	Fold Enrichment
GOTERM_MF_FAT	GO:0005524~ATP binding	106	11,84	5,84E-04	1,37
GOTERM_MF_FAT	GO:0032559~adenyl ribonucleotide binding	107	11,96	6,14E-04	1,36
GOTERM_MF_FAT	GO:0032553~ribonucleotide binding	126	14,08	9,05E-04	1,31
GOTERM_MF_FAT	GO:0032555~purine ribonucleotide binding	126	14,08	9,05E-04	1,31
GOTERM_MF_FAT	GO:0030554~adenyl nucleotide binding	110	12,29	1,20E-03	1,33
GOTERM_MF_FAT	GO:0000287~magnesium ion binding	40	4,47	1,44E-03	1,69
GOTERM_MF_FAT	GO:0000166~nucleotide binding	148	16,54	1,56E-03	1,26
GOTERM_MF_FAT	GO:0017076~purine nucleotide binding	129	14,41	1,65E-03	1,28
GOTERM_MF_FAT	GO:0001882~nucleoside binding	111	12,40	1,77E-03	1,31
GOTERM_MF_FAT	GO:0001883~purine nucleoside binding	110	12,29	2,01E-03	1,31
GOTERM_MF_FAT	GO:0060589~nucleoside-triphosphatase regulator activity	35	3,91	5,92E-03	1,62
GOTERM_MF_FAT	GO:0043492~ATPase activity, coupled to movement of substances	13	1,45	1,23E-02	2,26
GOTERM_MF_FAT	GO:0030695~GTPase regulator activity	33	3,69	1,28E-02	1,56
GOTERM_MF_FAT	GO:0005085~guanyl-nucleotide exchange factor activity	16	1,79	1,34E-02	2,01
GOTERM_MF_FAT	GO:0047485~protein N-terminus binding	10	1,12	1,47E-02	2,58
GOTERM_MF_FAT	GO:0030145~manganese ion binding	16	1,79	1,49E-02	1,98
GOTERM_MF_FAT	GO:0004672~protein kinase activity	44	4,92	2,56E-02	1,39
GOTERM_MF_FAT	GO:0042626~ATPase activity, coupled to transmembrane movement of substances	12	1,34	2,71E-02	2,10
GOTERM_MF_FAT	GO:0016887~ATPase activity	27	3,02	2,79E-02	1,54
GOTERM_MF_FAT	GO:0016820~hydrolase activity, acting on acid anhydrides, catalyzing transmembrane movement of substances	12	1,34	3,05E-02	2,06
GOTERM_MF_FAT	GO:0015631~tubulin binding	11	1,23	3,61E-02	2,10
GOTERM_MF_FAT	GO:0019899~enzyme binding	38	4,25	3,78E-02	1,39

Supplementary Table 3 | DAVID gene ontology analysis for the 959 synthetic lethal candidates.

Enriched significant molecular function terms obtained. $P < 0.05$.

			Transcriptome		Synthetic lethal screening		
**Probeset**	**Symbol**	**Gene name**	**Fold Change (BS-Ctrl$_{(BLM)}$ / BS-BLM)**	***P***	**Viability BS-BLM**	**Viability BS-Ctrl$_{(BLM)}$**	**Ratio BS-BLM/ BS-Ctrl$_{(BLM)}$**
201850_at	*CAPG*	capping protein (actin filament), gelsolin-like	1,63	1,93E-03	1,092	0,005	218,4
219387_at	*CCDC88A*	coiled-coil domain containing 88A	2,94	1,18E-03	1,537	0,048	32,02
219531_at	*CEP72*	centrosomal protein 72kDa	2,10	4,02E-04	1,747	0,014	124,79
229116_at	*CNKSR2*	connector enhancer of kinase suppressor of Ras 2	7,21	3,52E-04	0,740	0,012	61,67
226875_at	*DOCK11*	dedicator of cytokinesis 11	2,16	3,65E-04	0,774	0,024	32,25
212650_at	*EHBP1*	EH domain binding protein 1	1,56	4,31E-04	0,572	0,023	24,87
213900_at	*FAM189A2*	family with sequence similarity 189, member A2	8,74	8,27E-03	1,449	0,072	20,13
226886_at	*GFPT1*	glutamine--fructose-6-phosphate transaminase 1	1,64	2,62E-03	0,575	0,077	7,47
221901_at	*KIAA1644*	KIAA1644	5,15	2,34E-03	1,285	0,063	20,4
218311_at	*MAP4K3*	mitogen-activated protein kinase kinase kinase kinase 3	1,53	4,63E-03	1,563	0,083	18,83
**203929_s_at**	***MAPT***	**microtubule-associated protein tau**	**2,05**	**4,78E-02**	**0,627**	**0,039**	**16,08**
211105_s_at	*NFATC1*	nuclear factor of activated T-cells, cytoplasmic, calcineurin-dependent 1	4,61	2,07E-06	0,826	0,06	13,77
203038_at	*PTPRK*	protein tyrosine phosphatase, receptor type, K	1,51	8,01E-03	1,296	0,066	19,64
203020_at	*RABGAP1L*	RAB GTPase activating protein 1-like	1,56	2,00E-03	1,038	0,009	115,33
218137_s_at	*SMAP1*	small ArfGAP 1	1,56	6,58E-03	1,066	0,063	16,92
203217_s_at	*ST3GAL5*	ST3 beta-galactoside alpha-2,3-sialyltransferase 5	5,70	2,17E-05	0,907	0,001	907
213351_s_at	*TMCC1*	transmembrane and coiled-coil domain family 1	2,07	3,09E-03	1,076	0,013	82,77
224747_at	*UBE2Q2*	ubiquitin-conjugating enzyme E2Q family member 2	1,51	1,04E-04	0,589	0,004	147,25
229812_at	*USP48*	ubiquitin specific peptidase 48	1,57	1,46E-03	0,8	0,011	72,73
226148_at	*ZBTB44*	zinc finger and BTB domain containing 44	1,64	2,17E-03	1,285	0,016	80,31
203521_s_at	*ZNF318*	zinc finger protein 318	1,66	5,94E-03	1,196	0,026	46

Supplementary Table 4 | Synthetic lethal candidates upregulated in transcriptomic analysis of BS-Ctrl$_{(BLM)}$ cells relative to BS-BLM cells.

Category	GO term	Count	%	*P*	Fold Enrichment
**GOTERM_BP_FAT**	**GO:0051493~regulation of cytoskeleton organization**	**3**	**14,29**	**8,44E-03**	**19,89**
**GOTERM_BP_FAT**	**GO:0033043~regulation of organelle organization**	**3**	**14,29**	**2,05E-02**	**12,47**
GOTERM_BP_FAT	GO:0043242~negative regulation of protein complex disassembly	2	9,52	4,16E-02	43,99
**GOTERM_CC_FAT**	**GO:0044430~cytoskeletal part**	**4**	**19,05**	**3,33E-02**	**4,88**

Supplementary Table 5 | The enriched GO functions for the 21 upregulated genes identified with DAVID software.

siRNA	Sequence 5' to 3'	Species	Description	Reference
Non-targeting siRNA pool	UGGUUUACAUGUCGACUAA	Human	ON-TARGETplus SMART-pool	(7)
	UGGUUUACAUGUUGUGUGA			
	UGGUUUACAUGUUUUCUGA			
	UGGUUUACAUGUUUUCCUA			
siMAPT pool	UAGGCAACAUCCAUCAUAA			This work
	CACGGACGCUGGCCUGAAA			
	GGACACGUCUCCACGGCAU			
	AAGCUGACCUUCCGCGAGA			
siBLM pool	CUAAAUCUGUGGAGGGUUA			(7)
	GAUCAAUGCUGCACUGCUU			
	GGAUGACUCAGAAUGGUUA			
	GCAACUAGAACGUCACUCA			
siCDA pool	GGCAAUUGCUAUCGCCAGU			(7)
	CUUCAAAGGGUGCAACAUA			
	AGGCAAGUCAUGAGAGAGU			
	CCUACAGGGACUGGGCAAA			
siUBTF pool	UAACCAAGAUUCUGUCCAA			This work
	GGACCGUGCAGCAUAUAAA			
	CCAAUAAACGUAAGAGCAU			
	GAAGUUCCGUACAUUGACA			

qPCR primers		Sequence 5' to 3'	Species	Description	Reference
MAPT	F	GCTCATTAGGCAACATCCATC	Human	primer set sequences	This work
	R	GTCAGCTTGTGGGTTTCAATC			
CDA	F	CCCTACAGTCACTTTCCTG			(7)
	R	CGGGTAGCAGGCATTTTCTA			
BLM	F	CTGATGCCGACTGGAGGTG			(7)
	R	TGACAACAGTGACCCCAGGA			
B2M	F	CGCTCCGTGGCCTTAGC			This work
	R	GAGTACGCTGGATAGCCTCCA			
B-actin	F	CTGGAACGGTGAAGGTGACA			This work
	R	AAGGGACTTCCTGTAACAATGCA			
TBP	F	TGCACAGGAGCCAAGAGTGAA			This work
	R	CACATCACAGCTCCCCACCA			

ChIP-qPCR primers		Sequence 5' to 3'	Species	Description	Accession number	Reference
P1 (0.3)	F	TGTCAGGCGTTCTCGTCTC	Human	Ribosomal DNA Repeating Unit	U13369	(20)
	R	GAGAGCACGACGTCACCAC				
P2 (3.8)	F	GGATGCGTGCATTTATCAGA				(20)
	R	GTTGATAGGGCAGACGTTCG				
P3 (6.6)	F	GGTGGATCACTCGGCTCGT				This work
	R	GCAAGTGCGTTCGAAGTGTC				
P4 (11.9)	F	GAACTTTGAAGGCCGAAGTG				This work
	R	ATCTGAACCCGACTCCCTTT				
P1 (0.3)	F	TTGACGTACAGGGTGGACT				(19)
	R	GGAAGTTGTCTTCACGCCT				
P2 (3.8)	F	AAGCTGGCCGATCTGAATAA				(20)
	R	TTCCCAAGTCTGGTTGATCC				
P3 (6.6)	F	AGGTGTCCGTGTCCGTGT				(20)
	R	GGACAGCGTGTCAGCAATAA				

Supplementary Table 6 | List of siRNA sequences, and of qPCR and ChIP-qPCR primers used in the study.
